# Impacts of increasing levels of salt on intake, digestion, and rumen fermentation with beef cattle consuming low-quality forages

**DOI:** 10.1093/tas/txz111

**Published:** 2019-12-16

**Authors:** Hayley C White, Noah G Davis, Megan L Van Emon, Samuel A Wyffels, Timothy DelCurto

**Affiliations:** Department of Animal and Range Sciences, Montana State University, Bozeman, MT

## INTRODUCTION

Self-limited supplements are popular among beef cattle producers that use low-quality forage resources as a main feed source (DelCurto et al., 2000). Salt (NaCl) is the most common intake limiter because it is readily available, generally safe, and salt level can be modified to achieve the desired intake amount (Kunkle et al., 2000). Labor costs are therefore lowered because large amounts of supplement can be placed in a self-feeder and left in the pasture (Bowman and Sowell, 1997). However, daily individual intake of salt-limited supplement can be highly variable (Williams et al., 2018; Wyffels et al., 2018). This high variability of self-fed supplement intake between individuals can have negative effects on the profit of the producer by increasing costs (Bowman and Sowell, 1997), however little is known on the effects of high salt levels on the intake and digestion of low-quality roughages. One study evaluating the effects of supplemental salt on digestive parameters in growing beef cattle fed fescue hay, found that high salt diets altered rumen function, and observed increased acetate and acetate:propionate ratio and decreased valeric acid concentrations (Harvey et al., 1986). In general, research is limited and most studies evaluating the effects of salt on intake, digestion, or ruminal fermentation are specific to dairy cattle (Rogers et al., 1982; Wiedmeier et al., 1987) or beef steers consuming high concentrate diets (Meyer et al., 1955). Our research will provide more insight on how intake and digestion are affected by increasing salt levels in cattle fed low-quality diets.

The objectives of this study were to evaluate the impacts of supplemental salt levels on forage intake, water intake, dry matter (DM) digestibility, and rumen fermentation of beef cattle consuming high fiber, low-quality forages. We hypothesized that increasing levels of salt modifies rumen fermentation and digestion.

## MATERIALS AND METHODS

Experimental procedures described herein were approved by the Agriculture Animal Care and Use Committees of Montana State University (#2017-AA09). All animals used in this study were provided by the Montana Agricultural Experiment Station, and the study was conducted during the summer period at the Bozeman Agriculture Research and Teaching farm at Montana State University in Bozeman, MT.

Six Angus crossbred heifers [14 mo of age; 449 ± 24 kg body weight (BW)] were surgically fitted with a ruminal cannula (Bar Diamond, Inc. Parma, ID), housed in individual stalls, and randomly assigned to three supplemental treatments in dual 3 × 3 Latin square design. Two animals were assigned to each treatment per period to determine the impact of salt level on dry matter intake, water intake, DM digestibility, digesta kinetics and rumen fermentation. Salt treatments consisted of 1) control, no salt (CON), 2) 0.05% of BW salt (LOW), and 3) 0.1% of BW salt (HIGH). A protein supplement of 50% cracked corn and 50% soybean meal fed at 0.3% of BW was mixed with salt treatments resulting in a total supplement composition fed at 0.3%, 0.35%, and 0.4% for CON, LOW, and HIGH, respectively. Diets were formulated to meet or exceed nutritional requirements for yearling heifers gaining 0.5 kg/d (NAS, 2016). Chopped grass hay was used as the base ration and was provided daily at 120% of the average daily intake of the previous 3 d (Table 1). Before the start of the experiment, heifers were adapted to a salt-limited (25% salt) supplement for 14 d prior to the initiation of the trial. Each period included a 14-d diet adaptation, 6 d of sample collection, 1-d collection of rumen fluid samples for ruminal and microbial profiles. Feed refusals (orts) were collected daily, each animal’s daily consumption was calculated, and subsequent feed and supplement/salt treatment were provided at 0800 hours.

**Table:** Table 1. Nutritional quality of protein supplement and chopped grass hay fed to yearling heifers

Item	DM	TDN	CP	NDF	ADF
Supplement^1^					
Period 1	90.4	86	36.1	9.4	7.1
Period 2	90.6	84	31.9	10.4	5.5
Period 3	90.7	84	30.1	9.9	5.1
Hay					
Period 1	93.6	57	7.5	65.4	42.9
Period 2	96.4	58	7.3	63.1	41.2
Period 3	95.1	57	7.4	64.2	41.5

^1^Supplements were composed of 50% soybean meal and 50% corn.

During the 6-d collection period, feed, supplement, orts, and fecal output were measured for each individual animal. Daily water intake was measured by weighing disappearance corrected for evaporation. Feed, supplement, and ort samples were dried at 55 °C for 48 h and fecal samples were dried at 55 °C for 96 h in a forced air oven and ground to pass through a 1-mm screen using a Wiley mill. Feed samples were analyzed for DM, crude protein (CP), neutral detergent fiber (NDF), and acid detergent fiber (ADF) (Van Soest, 1967).

The effects of salt level on intake, water consumption, and digesta kinetics were analyzed using an analysis of variance (ANOVA) with a generalized linear model for a replicated Latin square design. The effects of salt level on volatile fatty acid (VFAs), pH, and ammonia were analyzed using ANOVA with generalized mixed models for a repeated measure analysis in a replicated Latin square design. Data were plotted and log-transformed if needed to satisfy assumptions of normality and homogeneity of variance. Statistical significance was accepted at an alpha of 0.05 and trends were considered between 0.05 and 0.10. All statistical analyses were performed in R (R Core Team, 2017).

## RESULTS AND DISCUSSION

**Figure 1.**
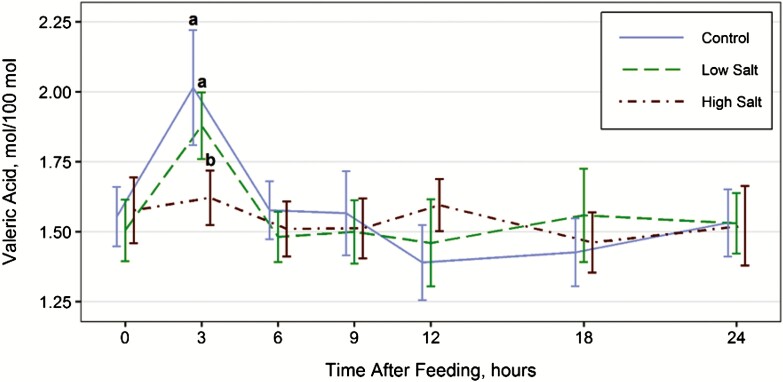
Effects of salt levels in supplement on concentration of valeric acid with an hour × treatment interaction (*P* = 0.01). Treatments include 1) CON, no salt, 2) LOW, 0.05% of BW, and 3) HIGH, 0.1% of BW. Means within hour that do not share a common letter differ (*P* < 0.05).

Influence of salt level on intake digestibility and rumen fill are listed in Table 2. Salt level had no influence on total forage intake (kg/d, *P* = 0.20) or DM digestibility (*P* = 0.75). However, intake expressed on a g/kg body weight tended (*P* = 0.07) to decrease with increasing levels of salt. Similarly DM fill tended to increase with increasing levels of salt or DM fill (*P* = 0.07). Increasing salt level increased water intake and liquid fill (*P* < 0.01) ranging from 50.8 to 60.4 and 62.7 to 73.1 l, respectively. Ruminal pH and ammonia levels both decreased with increasing salt level (*P* < 0.01; Table 3). However, total VFA concentrations were not influenced (*P* = 0.84) by salt levels averaging 82.8 mol/dl. Acetate molar concentration and the acetate:propionate ratio increased with increasing levels of salt (*P* < 0.01) suggesting decreasing fermentation efficiency with increasing levels of salt. In contrast, isobutyrate and butyrate concentrations decreased with increasing salt levels (*P* < 0.01). Valerate displayed a treatment × time interaction with treatment differences observed 3 h after feeding (*P* = 0.01), with control and low salt having a higher molar concentration compared to the high salt treatment (Fig. 1).

**Table 2.** Effect of increasing salt levels on intake, digestibility, and rumen fill of yearling heifers consuming low-quality forages

Item	Salt levels^1^			SEM	*P*-values		
	CON	LOW	HIGH		TRT^2^	LIN^3^	QUAD^4^
Forage intake, kg	9.5	9.6	9.2	0.14	0.20	0.16	0.22
Supplement intake, kg	1.0	1.2	1.4	0.01			
Total intake, kg	10.5	10.6	10.2	0.14	0.20	0.19	0.21
Forage intake, g/kg BW	25.6	25.3	24.3	0.33	0.06	0.03	0.42
Supplement intake, g/kg BW	2.7	3.2	3.7				
Total intake, g/kg BW	28.3	28.0	27.0	0.33	0.06	0.03	0.42
Water intake, l	50.8	53.1	60.4	1.30	<0.01	<0.01	0.15
DM digestibility, %	56.3	56.3	55.0	0.88	0.75	0.32	0.54
Liquid fill, l	62.7	69.9	73.1	1.09	<0.01	<0.01	0.18
DM fill, kg	10.8	11.5	12.1	0.33	0.07	0.23	0.86

^1^Salt levels include 1). CON, no salt, 2). LOW, 0.05% of BW, and 3). HIGH, 0.1% of BW.

^2^Treatment main effect.

^3^Linear preplanned contrast.

^4^Quadratic preplanned contrast.

**Table 3.** Effect of salt levels on ruminal parameters of yearling heifers consuming low-quality forages

Item	Salt levels^1^			SEM	*P*-values		
	CON	LOW	HIGH		TRT^2^	LIN^3^	QUAD^4^
pH	6.90	6.87	6.76	0.37	<0.01	<0.01	0.13
Ammonia, mg/dl	4.41	3.92	3.53	0.34	0.01	<0.01	0.84
Acetic, mol/100 mol	68.60	69.23	69.62	0.33	<0.01	<0.01	0.67
Propionic, mol/100 mol	16.66	16.71	16.66	0.17	0.94	0.97	0.73
Isobutyric, mol/100 mol	1.52	1.43	1.40	0.02	<0.01	<0.01	0.48
Butyric, mol/100 mol	9.92	9.42	9.16	0.23	<0.01	<0.01	0.31
Isovaleric, mol/100 mol	1.73	1.65	1.63	0.06	0.21	0.09	0.57
A:P ratio^5^	4.14	4.19	4.21	0.06	0.59	0.33	0.74
Total VFAs, mol/dl	84.16	82.55	81.51	3.90	0.84	0.56	0.94

^1^Salt levels include 1) CON, no salt, 2) LOW, 0.05% of BW, and 3) HIGH, 0.1% of BW.

^2^Treatment main effect.

^3^Linear preplanned contrast.

^4^Quadratic preplanned contrast.

^5^Acetate:propionate.

Studies have been conducted to evaluate the effects of salt on variables such as; intake, digestion, and ruminal fermentation, but these are specific to dairy cattle (Rogers et al., 1982; Wiedmeier et al., 1987), finishing beef cattle or sheep consuming high concentrate feeds (Meyer et al., 1955; Masters et al., 2005). Similar to the current study, increasing salt levels have been reported to decrease forage intake (Meyer et al., 1955; Masters et al., 2005), and increase water intake and liquid fill (Meyer et al., 1955; Rogers et al., 1982; Wiedmeier et al., 1987). Also similar to our study, high salt diets have decreased ruminal pH and ammonia and increased molar proportions of acetate, and acetate:propionate ratio although these were with high concentrate diets (Croom et al., 1982; Rogers et al., 1982; Wiedmeier et al., 1987). Although rumen fermentation characteristics were altered by salt intake in the current study, this did not affect overall digestibility. Despite the lack of information relative to salt and low-quality forages, impacts of salt on rumen function appear to be similar to research conducted with high concentrate, dairy and finishing rations.

## IMPLICATIONS

In agreement with a previous study (Harvey et al., 1986), our results demonstrate that high salt diets alter rumen function. In addition, our results similarly observed an increase in acetate, the acetate:propionate ratio, and a decreased valeric acid concentration with high salt levels. Results from this research provide additional information on how high salt diets can affect nutrient digestion in beef cattle consuming low-quality forages. Our research suggests that self-fed, salt-limited supplements may control intake, but may also result in lower intakes and less efficient rumen fermentation with beef cattle consuming low-quality forages.
